# The ladder of regulatory stringency and balance: an application to the US FDA’s regulation of bacterial live therapeutics

**DOI:** 10.1080/19490976.2025.2517377

**Published:** 2025-06-12

**Authors:** Moshe Maor, Hilit Levy Barazany, Ilana Kolodkin-Gal

**Affiliations:** a Lauder School of Government, Diplomacy & Strategy, Reichman University, Herzliya, Israel; b Scojen Institute for Synthetic Biology, Dina Recanati School of Medicine, Reichman University, Herzliya, Israel

**Keywords:** C difficile, FDA, fecal microbiota transplants, genetically modified organisms, probiotics, regulation

## Abstract

The three main types of live bacterial therapies – probiotics, fecal/microbiome transplants, and engineered bacterial therapies – hold immense potential to revolutionize medicine. While offering targeted and personalized treatments for various diseases, these therapies also carry risks such as adverse immune reactions, antibiotic resistance, and the potential for unintended consequences. Therefore, developing and deploying these therapies necessitates a robust regulatory framework to protect public health while fostering innovation. In this paper, we propose a novel conceptual tool – the *Ladder of Regulatory Stringency and Balance*—which can assist in the design of robust regulatory regimes which encompass medicine practices based not only on definitive Randomized Controlled Trials (RCTs), but also on meta-analyses, observational studies, and clinicians experience. Regulatory stringency refers to the strictness of regulations, while regulatory balance concerns the degree of alignment between the regulatory framework governing a technology and the actual risks posed by specific products within that technology. Focusing on the US regulatory environment, we subsequently position the three types of live bacterial therapies on the *Ladder*. The insight gained from this exercise demonstrates that probiotics are generally positioned at the bottom of the *Ladder*, corresponding to low-stringency regulation, with a proportionate regulatory balance. However, probiotics intended for high-risk populations are currently subject to low-stringency regulations, resulting in under-regulation. Our analysis also supports the conclusion that fecal microbiota transplants (FMT) for recurrent *Clostridium difficile* infection should be positioned close to but below the threshold for under regulation by the U.S. Food and Drug Administration (FDA), and we recommend improved donor screening procedures, preservation and processing, storage, and distribution. Our framework can serve as a scale to assess regulatory gaps for live bacterial therapies and to identify potential solutions where such gaps exist.

## Introduction

Live bacterial therapies are emerging as a treatment for a broad range of diseases. These therapies, which include probiotics, fecal/microbiome transplants, and engineered bacterial therapies, offer opportunities for targeted and personalized treatments across a wide range of diseases. Their potential to address complex health conditions that are poorly managed by traditional approaches underscores their importance in advancing medical innovation. However, the rapid development and deployment of these therapies are not without challenges. Chief among these are the risks of adverse immune reactions, antibiotic resistance, and the potential for unintended consequences, particularly in cases involving genetically modified organisms (GMOs).

The promise of live bacterial therapies is juxtaposed with the need for robust regulatory frameworks that can protect public health while fostering innovation. Ideally, the risks should also be evaluated using registries with standardized data, and randomized trials are crucial for ensuring safety. However, at times, the need arises to navigate regulatory decision-making under conditions of evidentiary uncertainty – specifically in contexts where medical interventions are supported by evidence that falls short of definitive RCTs, such as meta-analyses, observational studies and clinician’s experience.^1^ Striking the right balance between encouraging scientific advancement and ensuring safety, requires a nuanced approach to regulation-one that considers the unique risks and benefits associated with these therapies.^1^ Existing regulatory mechanisms often fail to account for the specificities of live bacterial products, leading to either overly restrictive policies that stifle innovation or overly lenient ones that jeopardize patient safety.

This paper introduces a descriptive-analytical regulatory framework to address these challenges, centered on a novel conceptual tool, *The Ladder of Regulatory Stringency and Balance*. This framework emphasizes two key dimensions, namely regulatory stringency, which refers to the strictness of regulations imposed on a technology, and regulatory balance, which highlights the alignment between the regulatory framework and the actual risks posed by specific products within that technology. Together, these dimensions provide a comprehensive perspective on regulation, helping to navigate the complexities of live bacterial therapies while avoiding the extremes of over-regulation and under-regulation.

By applying this framework to the regulation of live bacterial therapies in the United States, we aim to identify and address regulatory gaps in bacterial therapies currently overseen by the U.S. FDA. Through this analysis, we provide actionable insights into how regulatory systems can evolve to ensure both innovation and public safety, ultimately paving the way for the responsible advancement of live bacterial therapies.

## The Ladder

The *Ladder of Regulatory Stringency and Balance* is based on the two key dimensions, defined earlier. Regulatory stringency spans a spectrum ranging from low to high levels which corresponds to the FDA product classification manner, based on the claims made by manufacturers about their use, rather than their ingredients or other characteristics. The ‘regulatory framework’ for *low-stringency regulation* applies to dietary supplements. Probiotics, when used as dietary supplements, are regulated as ‘foods’ by the FDA’s Center for Food Safety and Applied Nutrition. Low stringency does not require premarket approval but mandates that the product is safe by law. The regulation is limited to validating label claims (without needing to submit them to the FDA) and post-marketing adverse event reporting. Manufacturers can self-determine that their products meet the “Generally Recognized as Safe” (GRAS) designation based on a history of prior use.^2^ The regulatory framework for *high-stringency regulation* is represented in the category of drugs and biologics and includes primarily requirements for an Investigational New Drug (IND) application to administer an investigational drug or biological product to humans.

The regulatory framework for *medium-level stringency* regulation has not been sufficiently addressed in scholarly literature. It covers products available for standard clinical use that do not require an IND application for their approved indications. This framework offers, for example, regulatory provisions for therapies categorized as exceptions to drug products used to treat infections unresponsive to standard therapies, some of which may still raise safety concerns for human subjects.

Regulatory balance spans a range from under-regulation, through proportionate regulation, to over-regulation. *Under-regulation* occurs when regulations are too lenient relative to the risk posed by the product. We place a product in this category if a pre-determined number of deaths following the use of a product or therapy in high-risk populations in hospital settings are recorded. This approach recognizes that the severity of adverse events can vary greatly, and for each technology, it is necessary to establish a figure that indicates increased risk. For probiotics administered to very preterm or very low birthweight (VLBW) infants (<1000 g), a product is categorized as under-regulated if one or more deaths following its use are recorded, coupled with more than 20 reported adverse events during a five-year period. This threshold aims to identify a level of harm significant enough to warrant stricter regulatory scrutiny without overreacting to isolated incidents. Although a single death may reflect rare, unpredictable events or specific circumstances, 20 reported adverse reactions recorded in specific high-risk populations suggest a systematic risk associated with the product’s use. This threshold strikes a balance between recognizing significant harm and avoiding premature conclusions about under-regulations based on insufficient evidence in the case of this technology. It covers cases where a therapy is among the best available for a particular disease, yet the intrinsic mechanism and active ingredient of the therapy are yet unknown. The threshold signifies a conservative approach to minimize regulatory error and conflicting interpretations regarding the use of this technology in specific high-risk populations.^3^ This approach aims to prevent instances where the FDA acts hastily when critical, negative information emerges, such as skipping the typically 60-day public comment period, as occurred with the FDA’s jurisdictional claims over human tissue transplants involving corneal lenticules (derived from the human cornea and used to correct vision problems) and dura mater (harvested from cadavers and used to patch brain sacs in living patients) following the detection of HIV/AIDS infection.^4^

A *proportionate regulation* is achieved when regulations are appropriately matched to these risks, with rules and oversight mechanisms proportionate to the hazards posed by the drug or treatment. In such cases, the regulatory framework neither overburdens nor under-protects, fostering an environment where public safety and innovation can coexist.

*Over-regulation*, on the other hand, occurs when the regulatory framework is excessively strict or unnecessarily adjusted relative to the actual risks posed by the drug or treatment. This can happen when regulations fail to distinguish between major and minor risks, leading to rules that are overly stringent or burdensome. A product or therapy is categorized here as over-regulated if no fatalities and fewer than a pre-determined number of adverse events are recorded. This approach acknowledges that the severity of adverse events can vary significantly, requiring each technology to have an established threshold for systematic risk and a defined time frame for assessing and comparing event frequency. For live bacterial therapies, a product is considered over-regulated if no fatalities and fewer than ten adverse events (e.g., serious non-lethal injuries or negative health outcomes) are recorded over a five-year period, despite being subjected to stringent regulatory requirements that severely limit accessibility, innovation, or affordability.

How does regulatory stringency change with regulatory balance? We have three regulatory frameworks that differ in their stringency levels: FMT at a high stringency level; FMT at a medium stringency level, for treating *Clostridioides difficile*infections unresponsive to standard therapies, provided informed consent is obtained; and probiotics at a low stringency level.

The high stringency level imposes a strict requirement for the submission of an IND application. In contrast, the medium and low stringency levels offer a range of regulatory alternatives. A high regulatory balance, or proportionate regulation, requires no changes in the regulatory stringency level pertaining to the health technology in question. However, a low regulatory balance presents more complex challenges and may manifest as either over-regulation or under-regulation.

In response to over-regulation, the initial course of action should be to adjust the regulatory stringency level associated with the technology by gradually relaxing the regulations in line with updated risk assessments. There may be exceptional cases where a shift to a lower stringency level is considered, particularly for investigational therapies, emergency situations (such as Coronavirus disease, COVID-19), or under specific regulatory discretion. Regarding the response to under-regulation, regulatory actions can initially be taken within the technology’s existing stringency level. However, as new information emerges about the declining safety for particularly vulnerable patients, it may be necessary to elevate the health technology to a higher-stringency regulatory level.

Let us now position FMT, genetically engineered microorganisms and probiotics, on the *Ladder*, considering the regulatory environment as of April 2025.

## Applying the Ladder to the FDA’s regulation of live bacterial therapies

**Figure 1.**
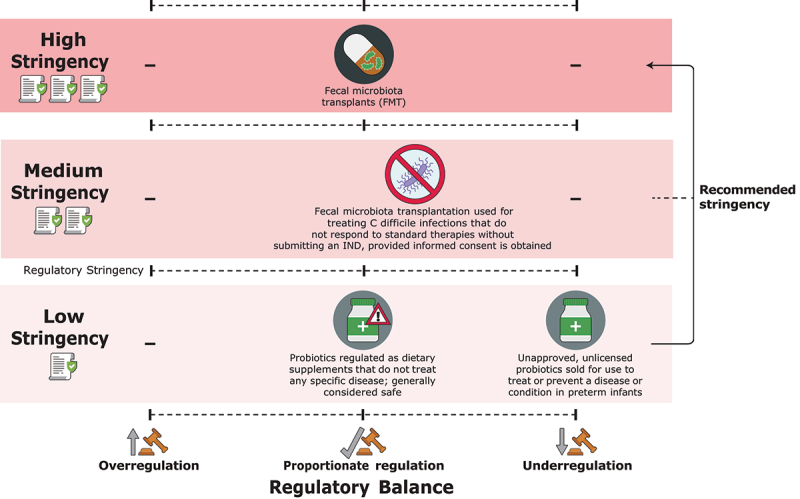
This manuscript covers three common forms of bacterial-based treatments: FMT, probiotics, and engineered bacterial therapies. Figure 1 illustrates their positions on the Ladder. Below, we describe each of these technologies and justify their respective positions on the *Ladder*. As of May 2025, no GMO based therapy was approved by the FDA. This is why no genetically engineered microorganism products are placed on the *Ladder*.

Biological based therapeutic technologies encompass the emerging use of living organisms in the treatment and diagnosis of diseases, affecting both humans and animals. In recent years, advancements occurred in various fields of biotherapy, including phage therapy (the use of viruses of bacteria to treat infections,^5,6^ and cell-based immunotherapy (CAR T-cell therapy, where the patients’ self-immune cells are engineered to treat their cancers).^7^ Within this growing category, bacterial cells are increasingly recognized as viable therapies for various diseases. This discussion covers three common forms of bacterial-based treatments: microbiome transplants, probiotics, and engineered bacterial therapies. Figure 1 illustrates their position on the Ladder. Below, we describe each of these technologies and justify their respective positions on the *Ladder*.

### Fecal microbiota transplantation (FMT)

The concept of ‘microbiota’ has its roots in the early 1900s, when scientists began to uncover the presence of bacteria, yeasts, and viruses in various regions of the human body, including the gut, skin, lungs, and oral cavity. The gut bacteria are essential for numerous functions, including the fermentation of food, protection against harmful pathogens, stimulation of the immune response, and production of essential vitamins.^8^ Dysbiosis in gut microbiota composition is linked to various diseases, including gastrointestinal diseases, neuropsychiatric disorders, autoimmune diseases, allergic conditions, and metabolic disorders.^9^

In 1958, Eiseman and his colleagues were the first to report the successful treatment of patients with *pseudomembranous colitis* using FMT.^10^ Later, in 2013, Els and colleagues conducted the first randomized controlled trial on the effectiveness of FMT. This study demonstrated that duodenal infusion of donor feces was significantly more effective in resolving symptoms of recurrent *C. difficile* infection (CDI), a bacterium that causes a chronic infection of the colon and becomes tolerant to antibiotics,^11^ compared to the use of antibiotics alone, a finding that was reproduced robustly by multiple research groups. Since then, numerous subsequent case studies have shown high cure rates for recurrent and refractory CDI with FMT.^12^ Moreover, systematic review by Chapman et al., determined that the success rates of FMT in treating CDI were between 83% and 100% with minimal adverse effects.^13^ The symptoms associated with treatment may include bloating, belching, abdominal cramps, discomfort, nausea, vomiting, increased flatulence, constipation, transient fever, urinary tract infections, self-limiting diarrhea, and irregular bowel movements. It is important to note that there have been instances of recurrent or refractory diarrhea following FMT, as well as worsening diarrhea in some cases. Notably, there were no reported instances of aspiration when FMT was delivered via the upper gastrointestinal route.^14^ In this meta-analysis, a case, where a patient experienced vomiting immediately after receiving FMT through a nasogastric tube was reported. Additionally, there was a single report of a mucosal tear and micro perforation after colonoscopic delivery of FMT (lower track).^14^

Currently FMT is being used experimentally to treat Inflammatory Bowel Diseases (IBD), such as Crohn and ulcerative colitis; functional Gastro Intestinal (GI) disorders, such as abdominal pain, and diarrhea; and non-GI disorders, with emphasis on clinical conditions that involve the immune system.^8^ Increasing evidence suggests that FMT from healthy patients can also reduce the burden of antibiotic resistance genes in chronic patients (although initial transfer of specific antibiotic resistance gens was also observed.^15,16^ The rationale behind FMT is that it can effectively restore a balanced intestinal microbiota. This process promotes the growth of beneficial bacteria and increases the abundance and diversity of symbiotic microorganisms in the gut. These microorganisms produce various bioactive substances, such as short-chain fatty acids (SCFAs), which are crucial for modulating immune responses.^8^ Overall, the efficiency of FMT is particular to the treated illness, as in the case of IBD, where it shows much less potency than for CDI.

This selective success highlights that the effectiveness of FMT can be challenged due to genetic, environmental, immunological, and microbial factors associated with various pathologies. Future clinical trials should consider patients’ characteristics, medications, and FMT timing, dosage, and frequency.^17^ Interestingly, there is a significant difference in the efficacy of fecal microbiota transplantation (FMT) depending on the delivery route, specifically between the lower GI and upper GI methods. The success rate for the lower GI route is 95% compared to 88% for the upper GI route.^14^

In the case of Crohn’s disease, a meta-analysis reviewed studies focused solely on one delivery method found that patients treated via the upper GI route experienced early clinical response rates ranging from 75% to 100%. In contrast, those receiving treatment through the lower route had response rates of only 30% to 58%. Furthermore, remission rates were notably higher for patients undergoing FMT via the upper GI route. After eight weeks, however, no specific delivery method demonstrated clear advantages in terms of patient outcomes.^18^

It is important to note that the associated risks differ between the upper GI route (which includes upper GI endoscopy, nasogastric tube, or naso-jejunal tube) and the lower GI route (such as colonoscopy, rectal tube, and colonic TET). Both procedures require careful administration.

From a regulatory perspective, FMT is classified as a drug in the United States and was therefore initially subject to stringent IND regulations, reflecting its novel nature and potential risks.^3^ As a result, this treatment is positioned at the highest regulatory stringency level on the Ladder.

An exception exists for FMT, which can be used for treating CDI that do not respond to standard therapies without the need for submitting an IND, provided informed consent is obtained. This protocol ensures that patients are informed of potential and known risks.^19^ It contains products administrated in two manners. In 2022, the FDA approved Rebyota,^20^ a broad-spectrum microbiota suspension for preventing recurrent CDI^21^ and in 2023, Vowst, the first oral fecal microbiota pill for the same indication.^22^ This treatment is therefore placed at the medium stringency (Figure 1).

Although the FDA has issued new screening guidelines in 2020^23^ after investigating two severe adverse events, as of 2020 there were five deaths from FMT.^24,25^ These rare fatalities highlighted a situation where, for FMT, the primary risk is the transmission of microorganisms, such as *Escherichia coli* (*E. coli)* and other drug-resistant bacteria present in donor material, despite compliant screening procedures. These incidents may have been the result of poor screening practices, not inherent safety flaws in FMT itself. Had screening protocols – such as those followed in Germany, France, Sweden, and other EU countries that regulate FMT as a drug^26^—been properly implemented, these events might have been avoided. In the broader EU context, we refer specifically to the stringent safety and quality requirements in compliance with Directives 2006/17/EC and 2006/86/EC implementing the European Union Tissues and Cells Directive (EUTCD) on the quality and safety of tissues and cells. In particular, Directive 2004/23/EC (EUTCD), specifies standards of quality and safety covering key aspects related to the stool donation, including screening, preservation, storage, and distribution.^26^

This suggests that, under current screening procedures in the U.S., FMT for treating *C. Difficile* infections should be positioned on the regulatory balance continuum closer to the under-regulation end. Note that the EU benchmark for the aforementioned under-regulation claim has been solidified on 17 July 2024, when the new EU SoHo regulation was published in the Official Journal of the EU (Regulation (EU) 2024/1938) covering standards of quality and safety for substances of human origin intended for human application and repealing Directives 2002/98/EC and 2004/23/EC.^27^ Currently, there are no uniform regulatory guidelines from European agencies concerning the screening of donors and donor stool, particularly regarding antibiotic-resistant genes (ARGs).^28,29^ Additionally, regulations vary among EU countries: Croatia, the Czech Republic, France, Germany, Ireland, Portugal, Spain, and Sweden classify fecal microbiota transplantation (FMT) as a medical product (similar to a drug). In contrast, Belgium and Italy regulate FMT under national legislation for tissues and cells, while Finland oversees fecal microbiota restoration (FMR) as a therapeutic intervention.^29^ Starting on 7 August 2024 and applying from 7 August 2027, the EU regulation specifically includes intestinal microbiota within its scope. It establishes high standards for the quality and safety of all substances of human origin (SoHO) intended for human use, as well as for the activities involving these substances, It aims to ensure a high level of health protection – particularly for SoHO donors, recipients, and offspring from medically assisted reproduction – by, among other things, enhancing the continuity of supply for critical SoHO.

Like many fields, the regulatory application of FMT could greatly benefit from synthetic biology. This involves creating artificially reproducible synthetic consortia that have been demonstrated to be safe. The precision microbiome approach might allow for a more uniform analysis of this therapy and could lead to reduced regulatory stringency for new applications. Although synthetic communities provide precise control over composition and enable manipulations such as strain dropouts and gene knockouts, they are typically low in complexity, usually consisting of fewer than 20 strains.^30^ This limitation reduces their ability to accurately model the biology of a native-scale microbiome. However, recent studies where community construction is achieved using robotic systems have shown the feasibility of developing more representative synthetic microbiomes that are better suited for therapeutic usage.^30,31^

### Genetically modified bacteria

Genetically engineered bacteria are heavily used in biotechnology and can be defined as bacteria that can effectively express heterologous proteins or altered molecular compounds for specific applications following genetic modification. The therapeutic potential of bacteria was first highlighted in the 19^th^ century by Coley, who demonstrated the effectiveness of inactivated bacterial mixtures in treating sarcoma.^32^ The most prominent bacteria used as a chassis for developing synthetic biology applications for biomedical industries are *E. coli*,^33^ and *B. subtilis*.^34^ Additionally, dozens of other bacterial species represented in the GI, including *Bifidobacterium* and *Lactobacillus* species are actively employed in healthcare and related research.^35,36^

Despite the widespread penetration of GMOs in biotechnology and industrial applications, the use of modified bacteria in medicine remains limited. Data from animal models has indicated exciting possibilities. For example, the genetically engineered beneficial bacterium *Lactobacillus* has demonstrated the capability to detect pathogen *Vibrio cholerae* in stool samples.^37^ As a representative example, the use of genetically modified bacteria versus tumors was most extensively studied in laboratory models. Specific bacteria, including *Salmonella* and *E. co*li, have shown a remarkable ability to colonize solid tumors, positioning them as potential candidates for targeted drug delivery and therapeutic production.^38^ In addition, in mice, a strain of *S. Typhimurium* that expresses transforming growth factor alpha (TGF-α) *pseudomonas* exotoxin has exhibited significant inhibitory effects on the growth of CT26, MC38, and 4T1 solid tumors.^39^ The broad repertoire of bacterial strains designed to target cancer is reviewed extensively here and includes but is not limited to engineering the bacteria-tumor interface, reprogramming the immune system and immuno-stimulation. Bacteria can be utilized in conjunction with imaging techniques such as Positron Emission Tomography (PET), Magnetic Resonance Imaging (MRI), and Focused Ultrasound (FUS) to enhance therapeutic outcomes. Drug-loaded nanoparticles can be physically attached to bacteria, allowing them to penetrate tumor depths that would otherwise be unreachable.^40^

Moreover, therapeutic strategies can also be based on interactions between living or replicating systems. For instance, bacteria can modify the tumor microenvironment (TME) to make it more favorable for oncolytic virus therapy.^41^ Additionally, synthetic consortia of bacteria can collaborate to induce predictable immune responses or diminish populations of bacteria that promote tumor growth.^42^ Lastly, CAR-T cells can be activated by bacterial adjuvants and programmed to respond to synthetic antigens released by bacteria.^40^ These multi-living system approaches can be broadened beyond oncology for other pathological conditions.

In addition to established genetic methods for targeting and treating diseases, engineered bacteria can be programmed for precise growth regulation. One strategy involves generating mutants of the enzyme D-alanine racemase, allowing growth only in the presence of D-alanine (a condition known as D-alanine oxothropy).^43^ Another approach is the introduction of a synthetic circuit that controls bacterial growth, activating it only in response to an external signal that does not interfere with mammalian cells or is a part of the culture media.^44^ Furthermore, microbial growth can be regulated by incorporating a suicide switch into the culture. This could involve a toxin that inhibits growth being constantly expressed alongside an anti-toxin that neutralizes the toxin, which is expressed only when growth is necessary.^45^ These strategies are particularly important for managing the potential spread of genetically modified strains from patients to the environment and mitigating any hazardous effects they may pose.

As of April 2025, no genetically engineered microorganism products have been approved by the FDA, raising some questions regarding the lack of clinical trials in this application. However, although the FDA has excluded vaccines from the definition of live biotherapeutic products (LBPs),^46^ it is important to highlight the fact that two vaccines are based on genetically modified bacteria―Vaxchora, and Vivotif. These vaccines were still required to undergo IND applications as part of the FDA standard regulatory process for approving any new drug or biological product, including vaccines. Vaxchora is the first FDA-approved Cholera vaccination in the US and is the first and only FDA-approved recombinant bacterial strain for use in human^47,48^ containing a live attenuated strain of *Vibrio cholera* (A live attenuated vaccine (LAV) is created by reducing or eliminating the virulence of the pathogen while keeping it viable.). This drug was approved in 2016 as an oral vaccine for cholera prevention in individuals aged 2 through 64 years who are traveling to cholera-affected areas.^48,49^ Vivotif, an attenuated strain *Salmonella typhi* Ty21a, was approved by the FDA in 1989 as an oral typhoid vaccine in 1989.^50,51^ In addition, the *Bacillus Calmette-Guérin* (BCG) vaccine based on the bacterium was approved in 1990 for the treatment of high-risk non-muscle invasive bladder cancer.^52–54^

The Ladder advanced here allows for clarity, and highlights the need for scalability. A future-oriented regulatory framework should introduce into the Ladder’s top regulatory stringency level a risk-based and transparent pathway tailored to the unique features and risks of the genetically modified bacteria. Alongside the requirement for an IND application before clinical trials, the FDA may develop a tiered risk assessment framework combined with a tiered regulatory stringency framework based, for example, on the type and extent of genetic modification, potential for replication and environment release, therapeutic context (e.g., immunocompromised patients). Further, lower-risk modifications (e.g., knockouts without antibiotic resistance genes) could undergo streamlined review, while higher-risk designs would require full regulatory scrutiny. A higher level of scalability could help strike a balance between fostering innovation and ensuring robust public health protections.

### Probiotics

The term ‘probiotic’ is derived from Greek and translates to ‘for life.’ The definition of probiotics has evolved over time, especially with the growing interest in viable bacterial supplements and a better understanding of how they work. Historically, one of the most widely accepted definitions was provided by Fuller, who described probiotics as “live microbial feed supplements that beneficially affect the host animal by improving microbial balance”.^55^ However, the current definition, established by the Food and Agriculture Organization of the United Nations and the World Health Organization, states that probiotics are “live microorganisms that, when administered in adequate amounts, confer a health benefit on the host.”.^56^

The effectiveness of probiotics in treating various medical conditions has been evaluated in multiple trials. A wide range of pathologies has been addressed with probiotics, including metabolic diseases, antibiotic-associated diarrhea, chemotherapy-associated diarrhea, *Clostridium difficil*e-associated diarrhea, constipation, respiratory tract infections, inflammatory bowel disease (IBD), urinary tract infections (UTIs), liver encephalopathy, periodontitis, depression, vaginosis, ventilator-associated pneumonia, pancreatitis, incidence of ventilator-associated pneumonia, and infections in hospitalized patients in intensive care units (ICUs).^57^

In a meta-analysis conducted by Rondanelli *et al*., the reviewed literature indicated that the therapeutic effects of probiotics are considered “evidence-based” only for antibiotic-associated diarrhea, *Clostridium difficile*-associated diarrhea, and respiratory tract infections. In other areas, meta-analyses often struggle to define the specific types and biological effects of probiotic strains and their outcomes in various disease.^58^ Several strain-specific factors influence the effectiveness of probiotics-based therapeutics. These factors include the strain’s origin, its viability, the formulation of the antibiotic, and the dosage used. Additionally, host-specific conditions such as the host’s genetics and immune response, the composition of their natural microflora, and their lifestyle also need to be carefully studied. This research is essential to enhance and optimize the use of antibiotic strategies.^59^ Moreover, a notable proof-of-concept study highlighted the lack of information regarding a potential therapy that examined the effects of probiotic treatment on post-antibiotic recovery of the mucosal microbiome in both humans and mice. This approach is often recommended by physicians after antibiotic treatment to enrich the gastrointestinal (GI) tract with beneficial microbiota. However, the evidence for its potential benefits remains to be fully determined. In their findings, Suez et al. discovered that multi-strain probiotics resulted in a delayed and incomplete recovery of the microbiome and the host transcriptome. In contrast, FMT led to a rapid and nearly complete recovery within just a few days.^60^

In total, the existing literature suggests that treatments using single beneficial bacterial species, or their combinations need further systematic research. However, these approaches offer clear advantages, particularly the targeted nature of the therapy compared with FMT, which focuses on specific bacterial strains or combinations of strains. Additionally, there has been extensive review of genetic engineering techniques applied to probiotic bacteria to enhance their efficacy and therapeutic potential.^61^.

From a regulatory perspective, the FDA has not approved any probiotic product for use as a drug or biological product in infants of any age. As noted earlier, probiotics are regulated by the U.S. FDA and the Federal Trade Commission (FTC) as dietary supplements, a subcategory of foods.

However, probiotics for high-risk population are placed here under low-stringency and under-regulation. The need for a cautious perspective on probiotics was recognized as early as 2008,^62–68^ although a causal link between probiotic use and an increased risk of infection has yet to be validated.^59,69^ While probiotics are widely marketed as dietary supplements to support gut health, some unapproved and unlicensed probiotics are also being sold for the treatment or prevention of diseases, including efforts to reduce the risk of necrotizing enterocolitis (NEC) in preterm infants. The fact the FDA is aware of that was directly-stated by the agency, as follows: “The FDA is aware that certain probiotic products used in hospital settings to prevent NEC have contributed to invasive disease, including one infant death in 2023, and have been associated with more than two dozen other reported adverse events in the United States since 2018”.^70^ It subsequently sent a letter to healthcare providers warning them about this risk and has issued two warning letters to companies for illegally selling their products for use in treating or preventing certain diseases in preterm infants.

Specifically, in September 2023, the FDA issued a Dear Health Care Provider (DHCP) Letter warning that preterm infants given probiotics may face a risk of invasive, potentially fatal infections caused by the bacteria or fungi contained in these products. The letter specifically described a case where a preterm infant, administered *Evivo (B. infantis*^71^ EVC001) with medium-chain triglycerides (MCT) Oil in a hospital setting, developed sepsis caused by *Bifidobacterium longum*, resulting in the infant’s death.^70^ Given that certain probiotic products used in hospital settings to prevent necrotizing enterocolitis have contributed to invasive disease, including one infant death in 2023, and have been associated with more than two dozen other reported adverse events in the United States since 2018,^70^ one can conclude that probiotics for high-risk population should be subject to medium-level regulatory stringency rather than the current low stringency regulation.

See text box 1 with a suggested regulatory requirements that can be applied individually or in combination to probiotics for specific high-risk populations.

**Text box 1: Applying Regulatory Framework for Probiotics**

The regulatory options on offer include: (1) requiring all probiotics for specific high-risk populations (e.g., preterm infants, critically ill patients) to undergo pre-market notification with the FDA and to provide safety data, a description of manufacturing processes, and evidence supporting the strain’s safety profile for its intended use to ensure regulatory oversight without requiring full clinical trials as with drugs; (2) requiring manufacturers to submit strain-specific evidence demonstrating that the probiotic is safe for the target population and has a documented mechanism of action for its structure-function claims to improve confidence in probiotic products while avoiding the high bar of drug-level RCTs; (3) requiring enhanced Good Manufacturing Practices (GMPs) standards specifically for probiotics, focusing on verifying the presence and viability of live organisms throughout shelf life; testing for contamination with harmful bacteria, fungi, or other organisms, and ensuring accurate labeling of colony-forming units (CFUs) to improve product quality and ensure probiotics meet consistent safety standards; (4) requiring probiotic manufacturers to monitor and report adverse events, particularly for high-risk populations in order to help identifying safety concerns in real-world use without imposing drug-level pharmacovigilance requirements; (5) requiring manufacturers to ensure that probiotic labels include specific strain(s) contained within the product, their documented health effects, minimum viable CFU count guaranteed at the end of shelf life, and appropriate usage recommendations and warnings for high-risk groups, to improve consumer understanding and reduce misuse or unrealistic expectations; (6) mandating that structure-function claims for probiotics be substantiated with peer-reviewed scientific studies, such as observational studies or meta-analyses, and submitted to the FDA for review before use to ensure that claims are truthful and based on credible evidence, enhancing consumer trust; (7) prohibiting the marketing of probiotics for use in specific high-risk populations without additional safety and efficacy data to reduce the risk of harm in vulnerable populations; (8) requiring stability testing to ensure CFUs remain viable at the labeled dose until the expiration date to ensure product efficacy and consistency for consumers, and (9) requiring that specific high risk populations or their guardians be provided with written information, the right to decline treatment, and a requirement for signed informed consent when such products are used.

## Conclusion

Live bacterial therapies, including probiotics, FMT, and genetically engineered microorganisms, represent a transformative advancement in modern medicine, offering unprecedented opportunities for targeted and personalized treatments. However, the challenges associated with their deployment underscore the critical need for robust and adaptive regulatory frameworks. The novel *Ladder of Regulatory Stringency and Balance* developed here provides a dual-dimensional approach to address these challenges, balancing regulatory strictness with proportionality to actual product risks.

By applying the *Ladder* to live bacterial therapies, we demonstrated its utility in identifying regulatory gaps and offering tailored solutions for each category of therapy, especially those positioned at the medium stringency level. Probiotics, while generally safe, require more stringent oversight when used in high-risk populations. FMT, though highly effective for conditions like *C. difficile* infections, benefits from a moderate regulatory balance to address its emerging applications. Genetically engineered microorganisms, still in the early stage of medical deployment, necessitate stringent oversight to ensure biosafety and mitigate risks associated with genetic modifications.

This framework emphasizes the importance of avoiding extreme over-regulation, which can stifle innovation, and under-regulation, which can jeopardize public safety. By addressing these gaps, the *Ladder* serves as a tool for advancing both public health and innovation in live bacterial therapies. It also highlights the need for continuous evolution in regulatory approaches as scientific understanding and therapeutic applications of these technologies expand.

While the *Ladder* offers a valuable framework for considering the regulation of live bacteria, it may suffer from certain limitations in its application and may require further refinement to effectively address the complexities of this emerging field. We identify six key issues. First, the classification of therapies involves subjective risk assessments. For example, the threshold for under-regulation of probiotics-one death and twenty adverse reactions in specific high-risk population―may not (and should not) be universally applicable across all types of live bacterial therapies. The same applies to the arbitrary definition of a ‘substantial period’ of five years for determining over-regulation. Second, the appropriate timeframe may vary depending on the specific product, its intended use, and the potential for long-term adverse effects. Third, the criteria for determining ‘serious non-lethal injuries or negative health outcomes’ can be explicitly defined in relation to a specific product. Fourth, the *Ladder* relies on rigid categories for regulatory stringency (low, medium, high) and regulatory balance (under-regulation, proportionate, over-regulation), whereas in reality, the risk-benefit profile of therapies can be complex and may not always fit neatly into these predefined categories. Fifth, some may criticize the framework for its over-simplification, because it primarily focuses on adverse events, such as deaths and serious injuries, as the primary indicator of risk. This may not adequately capture other important risks, such as the emergence of antibiotic resistance, environmental impact, or the potential for unintended consequences. Sixth, reliable and consistent data on these adverse events may be challenging to collect and analyze, particularly for rare events. In summary, the critique suggests that refinement and adaptation of the *Ladder* may be necessary to ensure its effectiveness and applicability across the diverse spectrum of these therapies.

Moving forward, the integration of synthetic biology, precision microbiome approaches, and advanced genetic engineering will likely redefine the landscape of live bacterial therapies. Policymakers and regulatory agencies must adapt to these advancements by leveraging frameworks like the *Ladder* to foster innovation while safeguarding public health. This dual commitment to innovation and safety will be essential in unlocking the full potential of live bacterial therapies to revolutionize medicine.

## Data Availability

All data required to interpret this manuscript are available within the manuscript
